# Persistent dysbiosis of duodenal microbiota in patients with controlled pediatric Crohn’s disease after resolution of inflammation

**DOI:** 10.1038/s41598-024-63299-y

**Published:** 2024-06-03

**Authors:** Rebecca Pierce, Ning-Jiun Jan, Pankaj Kumar, Jeremy Middleton, William A. Petri, Chelsea Marie

**Affiliations:** 1 Division of Infectious Diseases and International Health, Department of Medicine, University of Virginia School of Medicine, Charlottesville, VA USA; 2 Department of Biochemistry and Molecular Genetics, University of Virginia School of Medicine, Charlottesville, VA USA; 3 Division of Gastroenterology and Hepatology, Department of Medicine, University of Virginia School of Medicine, Charlottesville, VA USA

**Keywords:** Crohn's disease, DNA sequencing

## Abstract

Crohn’s disease is an inflammatory condition of the intestine characterized by largely unknown etiology and a relapse remission cycle of disease control. While possible triggers have been identified, research is inconsistent on the precise cause of these relapses, especially in the under-researched pediatric population. We hypothesized that patients in remission would have persistent microbial and inflammatory changes in small intestinal tissue that might trigger relapse. To this end, we analyzed intestinal biopsy samples from six patients with pediatric Crohn’s disease in remission and a control group of 16 pediatric patients with no evident pathogenic abnormality. We identified compositional microbiota differences, including decreases in the genera *Streptococcus* and *Actinobacillus* as well as increases in *Oribacterium* and *Prevotella* in patients with controlled Crohn’s disease compared to controls. Further, a histologic analysis found that patients with controlled Crohn’s disease had increased epithelial integrity, and decreased intraepithelial lymphocytes compared with controls. Additionally, we observed increased peripheral CD4^+^ T cells in patients with pediatric Crohn’s disease. These results indicate that markers of intestinal inflammation are responsive to Crohn’s disease treatment, however the interventions may not resolve the underlying dysbiosis. These findings suggest that persistent dysbiosis may increase vulnerability to relapse of pediatric Crohn’s disease. This study used a nested cohort of patients from the Bangladesh Environmental Enteric Dysfunction (BEED) study (ClinicalTrials.gov ID: NCT02812615 Date of first registration: 24/06/2016).

## Introduction

Crohn’s disease is an inflammatory condition of the intestine with a poorly defined etiology that includes genetic^1^, environmental^2^, and immune components^3^. While therapies exist to manage symptoms, there is no cure^4^ and patients typically fluctuate through periods of remission and relapse^5^. Over 25% of patients with Crohn’s disease are diagnosed before the age 20^6^ and diagnosis before age 40 is associated with more aggressive disease^7^. Pediatric Crohn’s disease can have life-long consequences such as growth failure or delayed puberty^7,8^ and treatments may include immunosuppressive therapy and extensive intestinal resection^9^.

Most of what we understand about Crohn’s disease comes from studies based on adult-onset disease, thus pediatric Crohn’s disease has been relatively understudied. The exact etiology of Crohn’s disease is unknown but dysbiosis of intestinal microbes is thought to play a role. It has been postulated that the balance between commensal and pathogenic microbes interacting with the host’s immune system contributes to Crohn’s disease onset^10^. Studies have consistently found that patients with Crohn’s disease have decreased alpha microbial diversity^11^. A few particular changes in taxonomic abundances have been associated with the dysbiotic state in Crohn’s disease, including a decrease in *Firmicutes* abundance in both patients with adult-onset^12,13^ and pediatric Crohn’s disease compared to age-matched controls^14^.

Despite these significant advances, previous studies have also shown inconsistent data on other taxonomic associations. Many factors such as lifestyle^15^, country of origin and geographic provenance^16^, diet^17,18^, and site of acquisition^19^ affect microbiota composition. Because the microbiota composition varies between locations along the gastrointestinal tract, samples taken from different locations may yield different taxonomic associations. For example, one study found lower abundance of *Faecalibacterium prausnitzii* in the stool of patients with Crohn’s disease^20^ while analysis of colonic mucosa biopsies samples found increased *F. prausnitzii* in patients with Crohn’s disease^21^. Previous studies have shown the anti-inflammatory properties of this bacterium^22^. Therefore, these mixed findings suggest the role of this bacteria is more complex than originally hypothesized from fecal studies alone. Many studies have analyzed fecal microbiota due to relative ease of acquisition compared to intestinal samples, which require invasive techniques. However, feces can have an uneven distribution of bacteria and may not be fully representative of the intestinal microbiota^23^. Aspirates more accurately describe the luminal microbiota and biopsies more accurately describe the mucosal tissue microbiota; however both are difficult to obtain^24^. The stage of disease at the time of study may also contribute to heterogenous results. Most studies have analyzed patients with active Crohn’s disease, which makes it difficult to deconvolute the microbial taxonomic differences due to Crohn’s disease versus Crohn’s disease-associated inflammation. Some studies have examined the differences between Crohn’s disease in remission with active Crohn’s disease, and have found that differences in gut microbial populations are shown to persist in remission when compared to controls^25^. However, few have focused on an exclusively pediatric cohort.

The purpose of this analysis was to identify differences in the small intestinal microbiota of patients with controlled pediatric Crohn’s disease. As Crohn’s disease is a chronic, treatable disease in which patients frequently suffer from a cycle of remission and relapse, we hypothesized that small intestinal dysbiosis in patients with pediatric Crohn’s disease could persist after remission and that persistent alterations could underlie the continued predisposition to loss of homeostasis. To test this hypothesis, we compared patients with controlled pediatric Crohn’s disease undergoing duodenal endoscopy to a group of pediatric patients who underwent endoscopy with no findings of pathogenic abnormality (referred to here as controls). We examined microbiota using 16S rRNA sequencing, pathological changes in tissue morphology and target proteins using immunohistochemistry (IHC) and circulating immune cell populations using flow cytometry.

## Methods

### Study design and participants

We analyzed a nested cohort of children from University of Virginia Hospital within the Bangladesh Environmental Enteric Dysfunction (BEED) study (ClinicalTrials.gov identifier NCT02812615). A complete description of the study design and procedures has been published^26^. Briefly, pediatric participants (age 1–17 years) were recruited from children undergoing esophagogastroduodenoscopy as a part of their medical care at the University of Virginia Health system. This cohort excluded any stunted children. A subset of children underwent esophagogastroduodenoscopy to monitor Crohn’s disease pathology and were selected as the study group (n = 6). We also identified a subset of children who underwent endoscopy and biopsy that had no pathogenic abnormality based on chart review and examination of small bowel histology. These children were selected as a control group (n = 16) for comparative analysis (Table 1, supplementary materials, supplementary methods for cohort selection).

**Table 1:** Clinical and histologic data from pediatric Crohn’s disease and control cohorts.

	Crohn’s disease (n = 6)	Controls (n = 16)
Clinical characteristics		
Female, percent (no./total)	66.7% (4/6)	50.0% (8/16)
Baseline BMI, mean (SD)	21.8 (3.2)	19.9 (3.2)
Age in years, mean (SD)	12.4 (3.0)	11.7 (4.2)
Antibiotic usage, percent (no./total)	0.00% (0/6)	6.25% (1/16)
Under active treatment for Crohn’s disease at time of biopsy, percent (no./total)	100% (6/6)	0% (0/16)
Crohn’s disease subtype		N/A
L1 (ileal)	33.3% (2/6)	
L2 (colonic)	16.7% (1/6)	
L3 (ileocolonic)	50.0% (3/6)	
Treatment type		N/A
Tumor necrosis factor inhibitors	66.7% (4/6)	
Prostaglandin production inhibitor	16.7% (1/6)	
Purine synthesis inhibitor	16.7% (1/6)	
Flares* (no. since biopsy)		N/A
0	1	
1	3	
2	1	
3 or more	1	
Histological scoring		
Epithelial detachment, mean (SD)	0.5 (0.2)	1.1 (0.5)
Chronic inflammation, mean (SD)	1.1 (0.7)	0.9 (0.4)
Eosinophilic infiltration, mean (SD)	0.1 (0.2)	0.0 (0.0)
Intraepithelial lymphocytes, mean (SD)	0.1 (0.2)	0.4 (0.4)
Villus architecture, mean (SD)	1.3 (1.0)	0.7 (0.6)
Intramucosal brunner glands, mean (SD)	1.2 (1.6)	2.2 (1.2)
Goblet cell, mean (SD)	0.3 (0.4)	0.5 (0.5)
Foveolar cell metaplasia, mean (SD)	0.3 (0.7)	0.3 (0.8)
Enterocyte injury, mean (SD)	0.2 (0.3)	0.3 (0.3)
Tissue fragment, mean (SD)	3.0 (2.5)	4.2 (3.3)
Lymphoid aggregates, mean (SD)	1.2 (2.2)	0.7 (1.2)
Paneth cell density, mean (SD)	0.3 (0.4)	0.5 (0.4)

Flares were defined as a PCDAI score above 10 after a period of remission.

### Patient sample collection and handling

A total of seven samples were taken from each patient; two duodenal aspirates, four endoscopic biopsies, and one blood sample were obtained from each pediatric patient undergoing duodenal endoscopy at UVA enrolled in a research study (IRB-HSR#19466). Two duodenal aspirates were analyzed for luminal microbiota composition by 16 s rDNA sequencing. One biopsy was allocated for RNA and DNA extraction to define host transcriptome (from extracted RNA) and the epithelial-associated microbiota (from extracted DNA). Two biopsies were formalin-fixed and paraffin embedded (FFPE). One of these FFPE biopsies was stained with hematoxylin and eosin (H&E) for histological scoring by a team of blinded study pathologists. The other FFPE biopsy was stained for various proteins by IHC. The last tissue biopsy was disassociated for immunophenotyping, which was also performed on peripheral blood mononuclear cells isolated from peripheral blood. All experiments were performed in accordance with IRB guidelines and informed consent was obtained from all participants and/or their legal guardians in accordance with the Declaration of Helsinki.

### 16 s rRNA sequencing and analysis

During endoscopy, both duodenal aspirates and biopsies were allocated for microbiota analysis. Duodenal aspirates were obtained in two forms. Existing pools of aspirate present in the duodenum were collected via suction (aspirate sample), then sterile saline (5 mL) was infused into the duodenum and aspirated (lavage sample). The use of saline lavage was intended to recover duodenal microbiota in patients without an existing pool of duodenal fluid. All duodenal aspirate samples were aliquoted and then flash-frozen in liquid nitrogen for bacterial DNA extraction and subsequent 16 s DNA sequencing. Biopsies were immediately placed in AllProtect (Qiagen) and stored at − 80℃ until nucleic acid extraction. Nucleic acid was extracted using the AllPrep RNA/DNA Kit (Qiagen), which extracted both the DNA and RNA. The extracted DNA was used to analyze host epithelial-associated microbiota while the RNA was used for transcriptomic analysis (supplementary materials, supplementary methods, for duodenal biopsy sample collection, RNA extraction, and RNA sequencing).

Bacterial DNA from small intestinal biopsies was amplified using V4 specific primers and indexed using Nextera XT Index Kit, which was then sequenced by Illumina via MiSeq sequencing^27^. A mock sample (ZymoBIOMICS) was included as a control. Sequence data was processed using the DADA2 pipeline^28^ R v. 4.0.3 to form amplicon sequence variants. Taxonomy was then assigned using Silva release v.138. The number of reads for each amplicon sequence variate was next normalized using ‘DESeq2’ package v. 1.28.1 to calculate the relative abundance of bacterial taxa^29,30^. This information was then analyzed using the ‘phyloseq’ package v. 1.32.0^31^. Two metrics of alpha diversity (Shannon–Weaver and Simpson) were computed using phyloseq^31^. Differences in alpha diversity between the microbiota of patients with pediatric Crohn’s disease and controls were measured using the Wilcoxon rank sum test. Beta diversity metrics (Bray–Curtis distance and principal coordinate analysis PCoA) were assessed using the R ‘vegan’ package v. 2.5–7^32^ and significance was measured using permutational multivariate analysis of variance (PERMANOVA). Differential abundance was measured using the ‘DEseq2’ package and assessed for significance using the Wald test. Analyses were designed to be similar to other previously published microbiome analyses for comparability^33^.

### Histological scoring

Biopsies were immediately fixed in formalin and embedded in paraffin until sectioning and further analysis. The tissue was sectioned and stained with H&E to highlight morphological changes in tissue. Three pathologists scored these H&E stained sections in the recently developed EED scoring index in a blinded fashion^34^. Briefly, epithelial detachment, intraepithelial lymphocytes (IELs), villus architecture, and goblet cell density are scored on a scale of 0–4, while chronic inflammation, eosinophilic infiltration, intramucosal Brunner’s glands, foveolar cell metaplasia, enterocyte injury, tissue fragments, lymphoid aggregates, and Paneth cell density were scored on a scale of 0–3. For all parameters, a score of zero is consistent with healthy tissue and a higher score represents increasing abnormality or damage. Scores from each pathologist were averaged to obtain a mean score for each feature. A composite score was obtained by summing the scores across histological features.

### IHC for specific protein markers

Biopsies were immediately fixed in formalin, embedded in paraffin, and stored at room temperature until processing. A tissue microarray was created with tissue cores from embedded biopsies and inserted into a recipient paraffin block. A microtome was then used to cut sections and then stained with various IHC protein markers (supplementary materials, Table S1). The surface area of staining for each marker was normalized by the epithelial area, which was quantified by keratin 18 (KRT18) staining. IELs were quantified by IHC as the total area of CD3 staining within KRT18 staining area. A Welch’s unpaired two-sample *t*-test was used to determine significant differences in these proteins between patients with pediatric Crohn’s disease and controls.

### Flow cytometry for local and circulating immune cell populations

For analyzing the local tissue-specific immune cell populations, biopsies were placed immediately into RPMI-1640, no phenol red (Thermofisher). Single lamina propria mononuclear cells (LPMCs) were isolated using Accumax (Innovative Cell Technologies) digestion for 12–20 min at room temperature. For analyzing circulating immune cell populations, venous blood (5 mL) was collected immediately prior to biopsy and the peripheral blood mononuclear cells (PBMCs) were isolated using Ficoll-Paque (Cytiva). Isolated LPMCs and PBMCs were immediately transferred to −80℃ in CoolCell (Corning) cell containers for 2 days before transfer to liquid nitrogen for storage until analysis. Immediately prior to staining, cells were thawed and stained with fluorochrome-conjugated antibodies (supplementary materials, Table S2) as previously described^35^. Samples were analyzed with the three-laser Cytek Aurora Borealis flow cytometer. All events were collected for LPMCs and at least 100,000 events were collected for PBMC samples. Immune cell populations were quantified as a percentage of the CD45^+^ or leukocyte population (supplementary materials Fig. S1 for representative gating strategy). Significant differences in these immune cell populations between those from patients with pediatric Crohn’s disease and controls were determined using Welch’s unpaired two-sample *t*-test.

### Ethics declaration

All experimental protocols were performed in accordance with relevant guidelines and regulations set by Ethical Review Committee of icddr,b (protocol no: PR-16007; Version 1.03; 1 March 2016), the Ethical Committee of Dhaka Medical College (DMC/ECC/2016/39); and Institutional Review Board for Health Sciences Research (IRB-HSR 19,466) of University of Virginia, Charlottesville. Informed consent was obtained from all participants or their legal guardians and all research was performed in accordance with the Declaration of Helsinki.

## Results

### Participant characteristics

Ninety children (age 1–17 years) were recruited for research biopsy collection from children undergoing esophagogastroduodenoscopy as a part of their medical care. Of these, 9 children were previously diagnosed with Crohn’s disease and were undergoing endoscopy to monitor clinical pathology and response to treatment. Three of the diagnosed patients had signs of active disease and were excluded from further analysis. The remaining 6 patients had no signs of active disease and comprised the pediatric Crohn’s disease cohort described here. A subsequent chart review of pediatric Crohn's disease activity index (PDCDAI) scores found that 5 of these 6 patients experienced at least one flair-up since their study biopsy despite maintaining consistent treatment (Table 1). A separate subset of children who underwent esophagogastroduodenoscopy and biopsy that had no pathogenic abnormality based on chart review and examination of small bowel histology were selected as a control group (n = 16) for comparative analysis (Table 1).

### Microbiota differs across luminal and epithelial sample types from the small intestine

**Figure 1:**
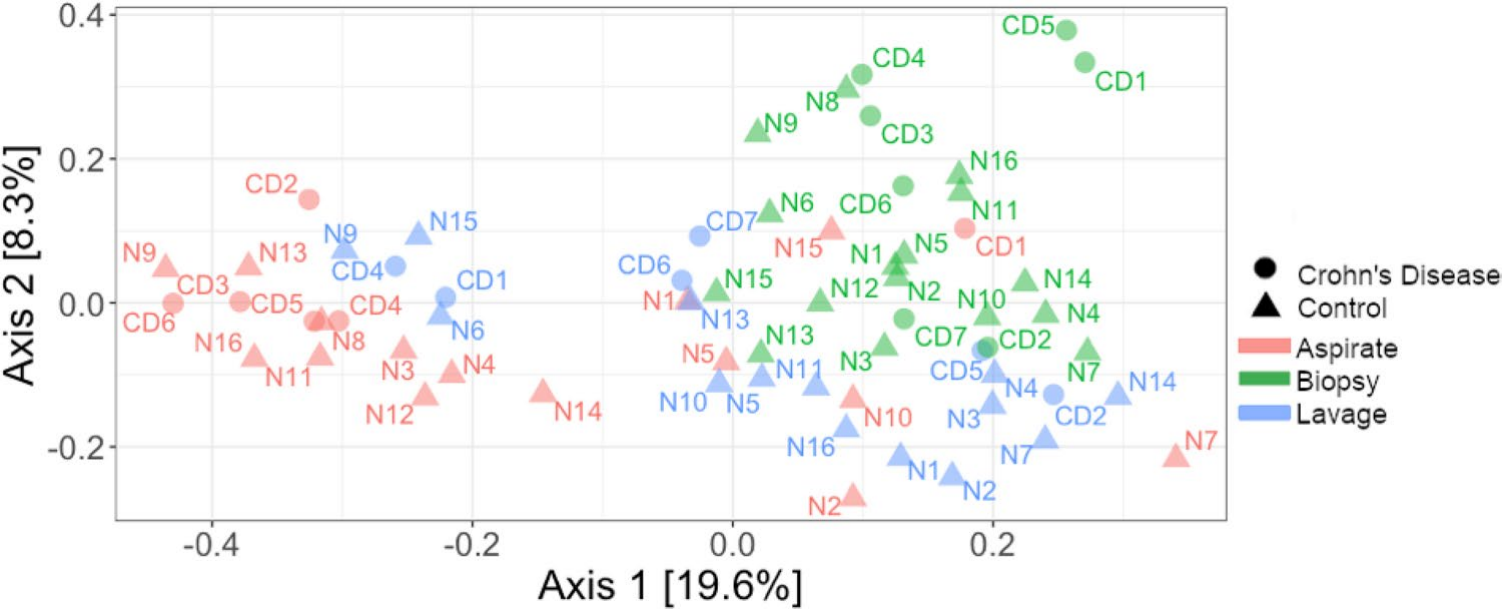
Microbiota composition is distinct between sample types (*p* = 0.001, PERMANOVA) PCoA axis 1 seems to be driven by differences between sample type (in order from left to right was aspirate, lavage, then biopsy). Bray–Curtis distance revealed that separation by sample type was significant (*p* = 0.001, PERMANOVA). Patient number is represented by CD# and N# for Crohn’s disease and controls respectively, while the colors indicated sample type.

We compared the epithelial-associated microbiota extracted from biopsy tissue to luminal microbiota extracted from duodenal aspirates and lavage samples. PCoA revealed distinct clustering by sampling site (Fig. 1). Quantitatively, the microbiota composition differed significantly between aspirate, lavage, and biopsy sample types (PERMANOVA, *p* = 0.001, Fig. 1). Aspirate and lavage samples were distinct while biopsy samples were grouped closely on axis 1, implying that our epithelial-associated biopsy samples had less variability related to sampling.

### Microbiota composition is distinct in controlled pediatric Crohn’s disease

**Figure 2:**
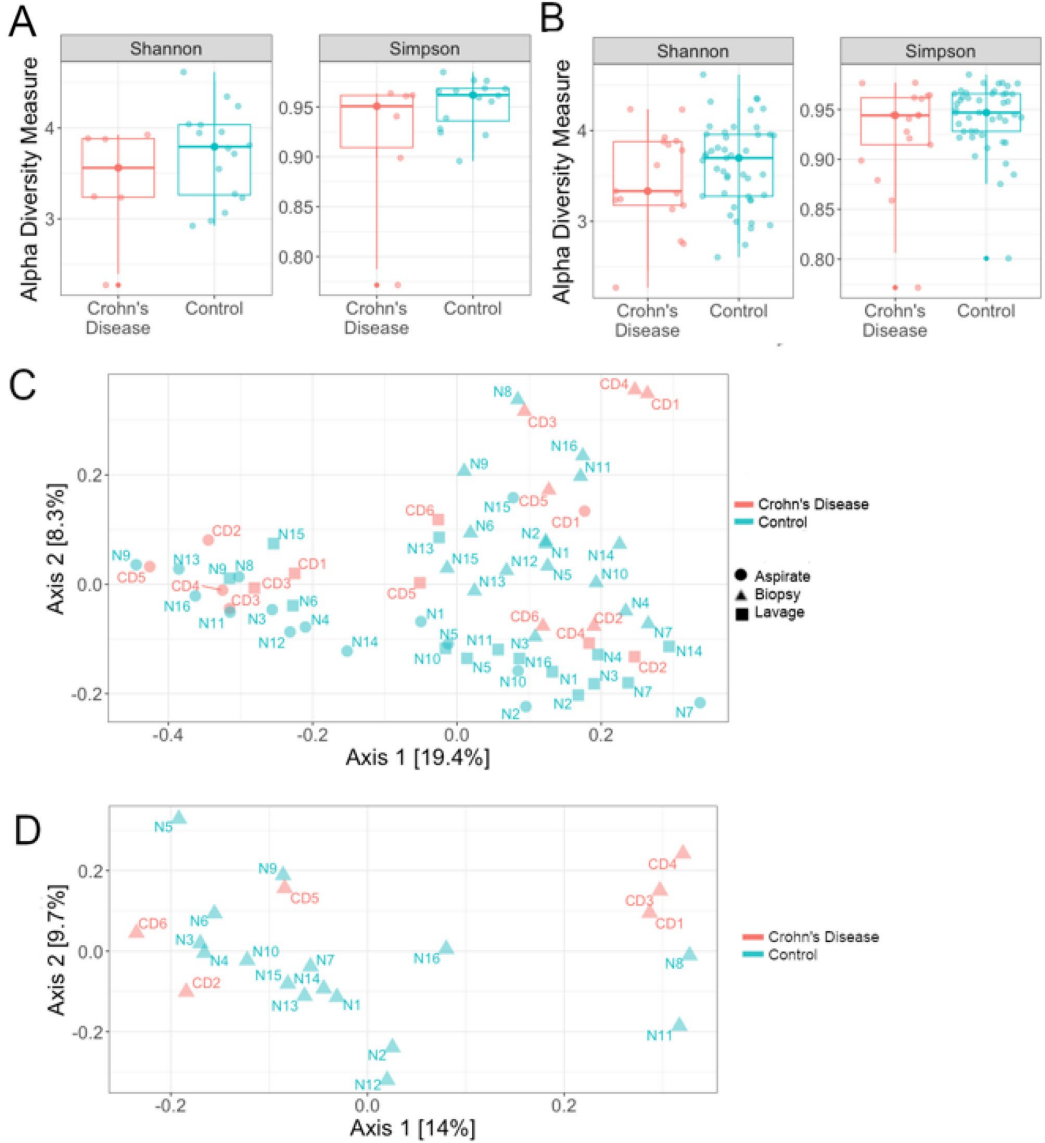
Reduced alpha diversity and microbial differences in pediatric Crohn’s disease. (**A**) Epithelial-microbiota alpha diversity was decreased in Crohn’s disease however this did not reach significance (*p* = 0.32 (Shannon) and *p* = 0.28 (Simpson), Wilcoxon unpaired signed rank test. In both panels, the line represents the median and whiskers indicate the interquartile range (IQR). See supplementary materials Table S4 for a statistical summary. (**B**) Alpha diversity decreased in Crohn’s disease compared to controls across both luminal and epithelial microbiota (Simpson: *p* = 0.26, Shannon (left) *p* = 0.24, Wilcoxon unpaired signed-rank test). (**C**) PCoA of both luminal (aspirate and lavage) and epithelial (biopsy) sample types identified clustering by Crohn’s disease status along axis 2. The separation in Bray–Curtis distance between patients with controlled Crohn’s disease and controls was statistically significant (*p* = 0.08, PERMANOVA). (**D**) PCoA of epithelial-associated (biopsy) microbiota samples also indicate separation according to Crohn’s disease status in axis 2 (*p* = 0.17, PERMANOVA).

The alpha diversity of the duodenal epithelial microbiota was slightly decreased in patients with Crohn’s disease (Fig. 2A). The median Shannon diversity was 0.23 lower while the median Simpson diversity was 0.01 lower in patients with Crohn’s disease compared to controls. The alpha diversity of the combined (luminal and epithelial) microbiota was also slightly decreased in patients with Crohn’s disease relative to controls, with the medians 0.37 lower for Shannon diversity and 0.01 lower for Simpson diversity, respectively (Fig. 2B). Differences in beta diversity in patients with pediatric Crohn’s disease relative the control group were distinct using the Bray–Curtis distance metric across all sample types (*p* = 0.08, PERMANOVA, Fig. 2C). Aspirate and lavage samples grouped closely on axis 2 while biopsy samples were more variable (Fig. 2C), implying that the epithelial-associated microbiota composition of biopsy samples had more ability to discriminate by Crohn’s disease status. Subsequent compositional microbiota analyses focused on the epithelial-associated biopsy samples. PCoA of biopsy samples alone revealed groupings along axis 1 related to Crohn’s disease status, however these differences were not significant *p* = 0.17, PERMANOVA, Fig. 2D).

### Differential community composition of epithelial microbiota in controlled Crohn’s disease

**Figure 3:**
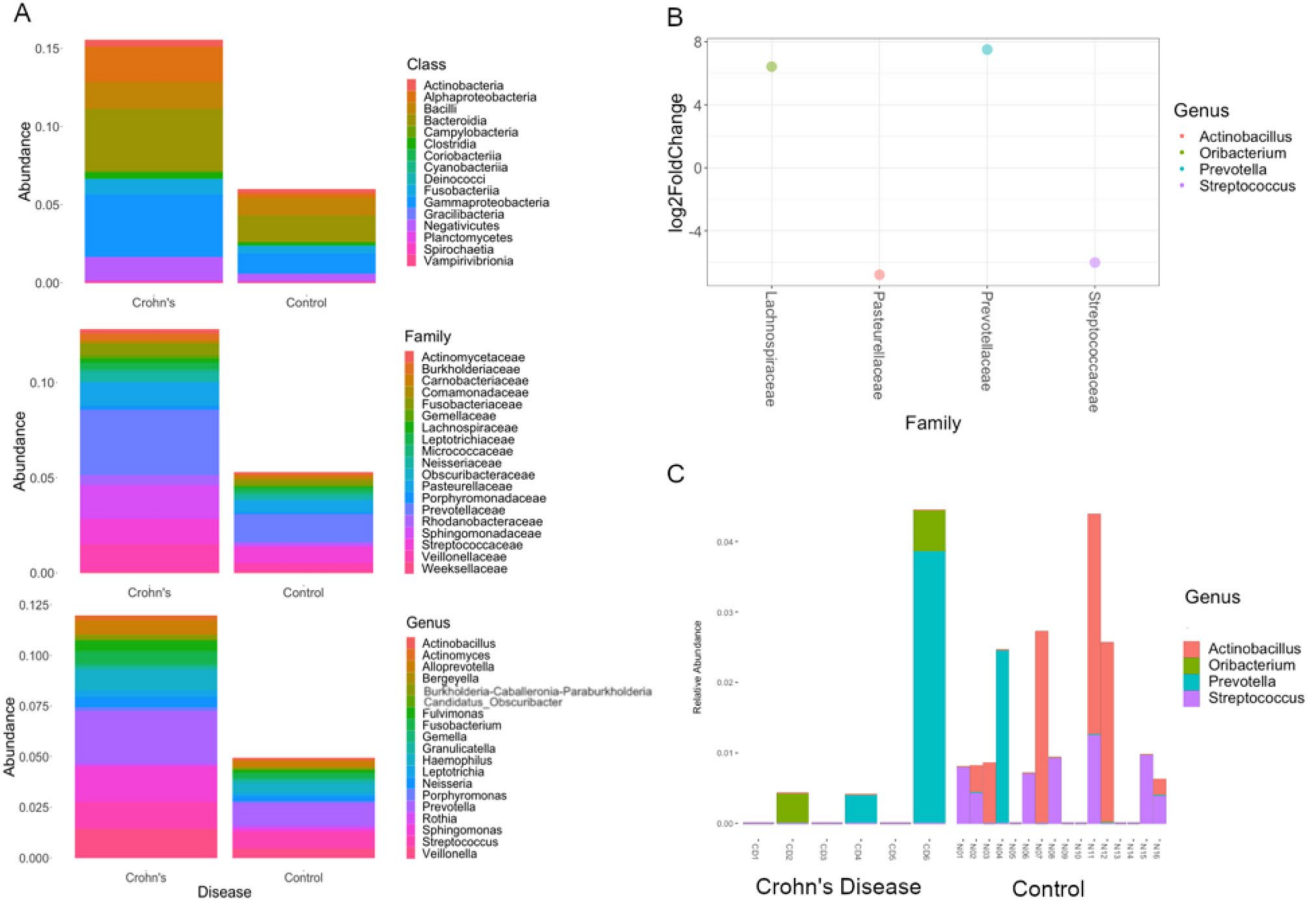
Pediatric Crohn’s disease shows differential community composition in epithelial-associated microbiota. (**A**) Crohn’s disease shows increased relative abundance for the top 20 genera compared to control. The average relative abundances by patient, for the top 20 genera are shown by class, family, and genus stratified by Crohn’s disease and control. (**B**) Pediatric Crohn’s disease showed deficiency and overabundance of specific bacterial taxa. Differential community composition in the epithelial microbiota in Crohn’s disease is characterized by increased *Prevotella* and *Oribacterium* and decreased *Actinobacillus* and *Streptococcus*. Plot shows taxa level differences between pediatric Crohn’s disease and control in epithelial-associated microbiota abundance. Differences with a |log_2_fold change|> 6 and *p* ≤ 0.05 by the Wald test are displayed (supplementary materials, Table S3). (**C**) Pediatric Crohn’s disease showed deficiency and overabundance of specific bacterial taxa that varied between patients.

To further explore differential abundance, we analyzed taxa-level differences in epithelial-associated microbiota of patients with controlled Crohn’s disease compared to controls. The top 20 most abundant classes, families, and genera showed higher relative abundances in patients with pediatric Crohn’s disease compared to controls (Fig. 3A).We identified two genera, *Actinobacillus* (log_2_fold change = − 6.8, *p* = 0.03, Wald test) and *Streptococcus* (log_2_fold change = − 6.0, *p* = 0.02, Wald test), that were significantly less abundant in patients with Crohn’s disease compared to controls (Fig. 3B, supplementary materials, Table S3). *Actinobacillus* and *Streptococcus* were absent in all patients with Crohn’s disease (Fig. 3C). Two genera, *Oribacterium* (log_2_fold change = 6.4, *p* = 0.05, Wald test) and *Prevotella* (log_2_fold change = 7.5, *p* = 0.02, Wald test), were increased in patients with Crohn’s disease (Fig. 3B, supplementary materials, Table S3). *Oribacterium* was absent in all controls (Fig. 3C).

### Crohn’s disease is associated with increased epithelial integrity and lower IELs

**Figure 4:**
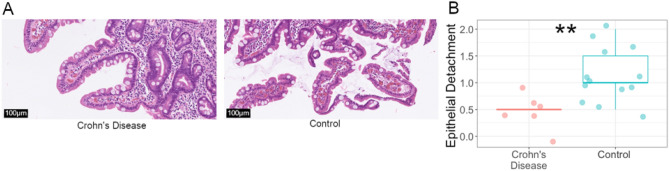
Decreased epithelial detachment in biopsy samples of patients with Crohn’s disease compared with controls (**p = 0.006). (**A**) Example biopsy tissue image shows decreased epithelial detachment in Crohn’s disease (left, epithelial detachment score of 0) compared to a control patient (right, epithelial detachment score of 3). Epithelial detachment was scored by three independent pathologists on a scale from 0 to 3 then averaged. Both patients had identical tissue fragmentation scores (= 1). (**B**) Epithelial detachment was significantly decreased in the samples from patients with Crohn’s disease compared to controls (**p = 0.006, Welch’s unpaired two sample *t*-test). The epithelial detachment was scored on a scale from 0 to 3 with 0 corresponding to no detachment. The line represents the median and whiskers indicate IQR. Note that these overlapped because Crohn’s had an IQR of 0 and the 25% quartile of the controls was equal to their median. See supplementary materials, Table S4 for a descriptive statistical summary.

**Figure 5:**
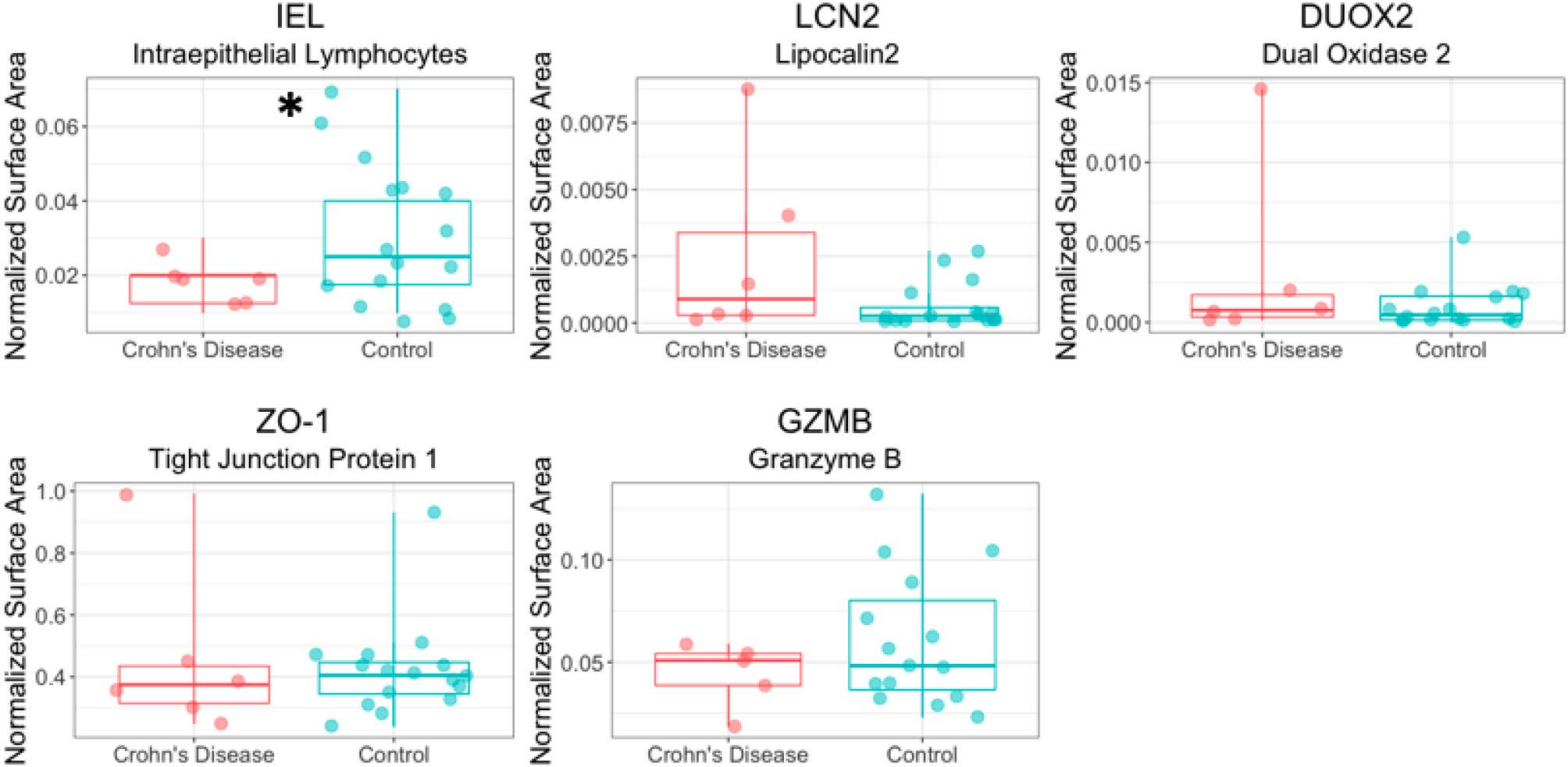
Intraepithelial lymphocytes are significantly decreased in duodenal epithelium in patients with Crohn’s disease *p = 0.05 (Welch’s unpaired two-sample *t*-test). Lipocalin2, dual oxidase 2, tight junction protein 1, and granzyme B did not significantly differ between Crohn’s and controls (Welch’s unpaired two-sample *t*-test). Stained marker area normalized by tissue surface area. The line represents the median and whiskers indicate IQR. See supplementary materials Table S4 for a statistical summary.

We compared histological scores and identified decreased epithelial detachment in patients with Crohn’s disease compared to controls (Fig. 4, *p* = 0.006, Welch’s unpaired two-sample *t*-test). All other differences in histological features between Crohn’s disease and control patients were statistically insignificant (Table 1). In examining quantitative IHC we found a significantly lower abundance of IELs in controlled Crohn’s disease compared to controls as measured by the CD3 stained area within the intestinal epithelium (Fig. 5, *p* = 0.05, Welch’s unpaired two-sample *t*-test). All other differences in the examined IHC epithelial markers were not statistically significant (Fig. 5, supplementary materials Table S4). In combination, these observations found that patients with controlled Crohn’s disease had enhanced epithelial integrity and decreased IEL infiltration relative to the control group.

### Increased peripheral CD4^+^ T cells in controlled Crohn’s disease

**Figure 6:**
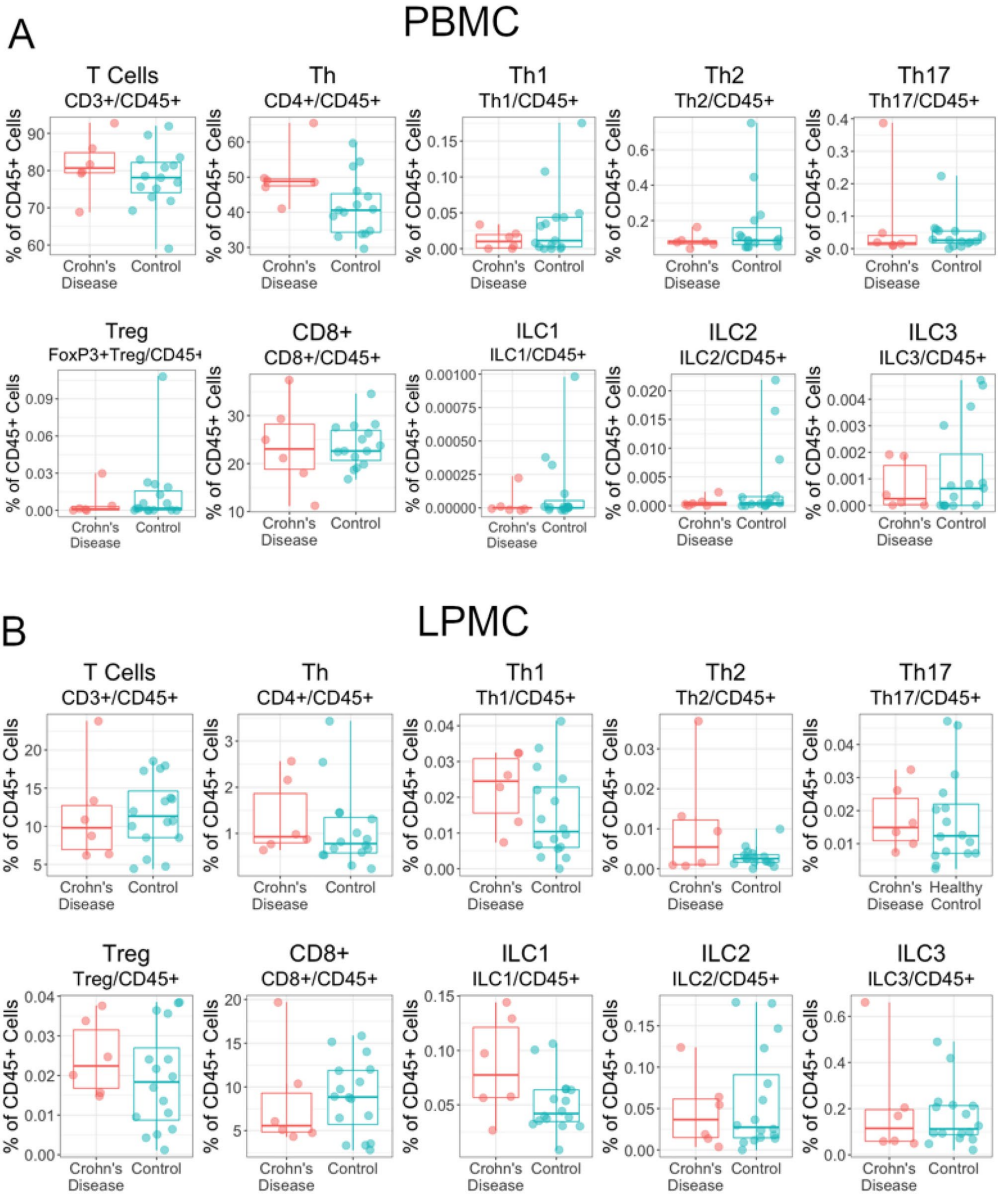
Circulating CD4^+^ T cells were increased in controlled pediatric Crohn’s disease (**A**). (**A**) Box plots of relative abundance of immune cells from PBMCs by high-dimensional flow cytometry. CD4^+^ T cells were increased in Crohn’s disease,* p* = 0.06 (Welch’s unpaired two-sample *t*-test). (**B**) Box plots of relative abundance of immune cells in LPMCs by high-dimensional flow cytometry. There were no significant differences detected in LPMCs between Crohn’s disease and controls. In both panels, the line represents the median and whiskers indicate IQR. See supplementary materials Fig. S1 for representative gating and Table S4 for a descriptive statistical summary.

PBMC and LPMC populations were analyzed by flow cytometry (Fig. 6). Peripheral CD4^+^ T cells were increased in patients with Crohn’s disease compared to controls (Fig. 6A, *p* = 0.06, Welch’s unpaired two-sample *t*-test). CD4^+^ T cells were also increased in LPMC preparations of patients with Crohn’s disease relative to controls; however, this finding was not significant (Fig. 6B, *p* = 0.80, Welch’s unpaired two-sample *t*-test). CD8^+^ T cells in the lamina propria were lower in controlled Crohn’s disease (Fig. 6B), consistent with the observation of decreased IEL (Fig. 5) but this did not reach significance. CD4^+^/CD8^+^ and Th1/Th2 ratios were also not significantly different in patients with Crohn’s disease compared to controls in either LPMCs or PBMCs (data not shown).

To follow up on our observation of limited changes in immune cell population in patients with Crohn’s disease compared to controls, we analyzed bulk RNA-seq data from small intestinal biopsies comparing Crohn’s disease to control patients. This analysis did not identify any genes between Crohn’s disease and the control group that met the criteria for significantly differential expression (*p* adj < 0.05, Wald test, supplementary materials, Table S5). A principal component analysis of the top 1000 most variable genes across groups also did not demonstrate any clustering according to Crohn’s disease state (supplementary materials, Fig. S2A). When examining gene expression of 130 known epithelial integrity genes we found no apparent trends toward altered expression in patients with controlled Crohn’s disease relative to controls (supplementary materials, Fig. S2B) including *MUC1* and *MUC4* (supplementary materials, Fig. S2C) which have previously been shown to remain dysregulated in controlled Crohn’s disease^25^.

## Discussion

Crohn’s disease relapse is poorly understood, especially in pediatric population. We hypothesized that microbial and inflammatory changes that persist during symptomatic remission might act as potential triggers for relapse. Congruent with previous research, the small intestinal microbial community in patients with pediatric Crohn’s disease was distinct from those of controls (Fig. 2) including significant differences in several specific epithelial-associated bacterial taxa (Fig. 3). We initially hypothesized that these alternations in the epithelial-associated microbiota would be associated with subtle inflammatory changes in small intestinal tissue. Surprisingly, controlled disease was associated with less inflammation as measured by increased epithelial integrity (Fig. 4) and lower IELs (Fig. 5); and there was no evidence of increased inflammatory infiltrates by flow cytometry (Fig. 5) or altered gene expression by RNA-seq (supplementary materials, Table S5 and Fig. S2). From these results, we concluded that while disease remission was marked by resolved tissue inflammation, a degree of microbial dysbiosis remained.

Decreased microbial diversity has been observed in active pediatric Crohn’s disease^33^ but the impact of microbial diversity during remission is less clear. We found no significant differences in alpha diversity in controlled disease, but marked differences in beta diversity (Fig. 2C), including increased *Prevotella* and *Oribacterium* and decreased *Lactobacillus* and *Streptococcus* in controlled disease (Fig. 3B). Recent work found that remission in response to dietary therapy with improved measures of intestinal permeability was accompanied by increased abundance of *Lachnospiraceae*^36–38^. This is consistent with our finding of increased *Oribacterium* (Class: Clostridia, Family: *Lachnospiraceae)* in patients with controlled Crohn’s disease and dovetails with our observation of enhanced epithelial integrity in effectively controlled disease (Fig. 4). *Oribacterium* was initially characterized as an oral bacteria^39^, and recent evidence suggests greater transmission of oral bacteria to the gastrointestinal tract than previously appreciated^40^. Oral pathobionts have been found at higher rates in the stool of patients with IBD compared to controls^41^. Previous findings have also shown that increased oral bacteria can facilitate overall dysbiosis of the gut, consistent with our results^42^.

The genus *Prevotella* has been associated with inflammation^43^ and increased *Prevotellaceae* was observed in duodenal biopsies from pediatric patients with active Crohn’s disease^44^. However, other studies have shown a decrease or even complete depletion of *Prevotella* in pediatric IBD^45–47^. In this study, *Prevotella* was increased in abundance in controlled Crohn’s disease (Fig. 3B) suggesting *Prevotella* alone is not sufficient to induce tissue inflammation. Our study also found decreased *Pasteurellaceae* (Fig. 3B) and decreased *Actinobacillus* (family *Pasteurellaceae*) (Fig. 3C) were associated with controlled pediatric Crohn’s disease, which contradicts finding from active pediatric disease^48^. As *Actinobacillus* in pediatric Crohn’s is not well documented this may be an area for future study. Similarly, previous studies have found conflicting results for *Streptococcus* with some studies showing significant increases^46,49–53^ and others finding decreases^54,55^ in pediatric IBD. These differences may be attributable to differences in disease state, or differences in species level composition that could be investigated further by metagenomics. As epithelial-associated samples are difficult to acquire for children, a strength of our analysis is the inclusion of this sample type. However, due to differences in fecal versus epithelial-associated samples, this sampling strategy may also complicate comparisons with other studies.

We hypothesized that epithelial-microbial dysbiosis would be accompanied by histological signs of a disrupted epithelial barrier. To the contrary, we found evidence of increased intestinal barrier function in controlled disease as measured by both decreased epithelial detachment (Fig. 4) and decreased IELs (Fig. 5). Data regarding IEL in Crohn’s disease is limited but previous studies have shown normal IEL counts^56^. In our controlled disease cohort, these levels may be further suppressed due to treatment with anti-inflammatory drugs and/or TNF-antagonists (Table 1). It is notable that in the absence of inflammation characteristic of active Crohn’s disease, we still observed microbiota composition differences compared to the control group suggesting that bacterial dysbiosis persists even when inflammation and epithelial barrier are improved by treatment.

Flow cytometry analysis found increased peripheral CD4^+^ T-cells in patients with Crohn’s disease compared to controls. Higher CD4^+^ T cells may reflect a persistent or heightened response to the intestinal microbiota that could contribute to initiating disease flares (characterized by increased PCDAI score), consistent with previous findings that have documented the role of microbiota dysbiosis in changes to T cell homeostasis^57^. While IHC identified differences in IELs, we did not observe significant alterations in lamina propria T cells by flow cytometry. However, we did observe a statistically insignificant increase in CD8^+^ LPMCs consistent with the increased IEL in the epithelium (Fig. 6B). Flow cytometry does not differentiate IELs from lamina propria T cells and IHC and flow cytometry were conducted on different biopsy samples so this could also reflect biopsy-level variation. Also, in flow cytometry, CD3^+^ cells were normalized to total CD45^+^ cells and did not include an additional marker to define IELs. IELs were defined as the CD3 stained area within the KRT18 stained area demarking the epithelium by IHC, which is a more accurate method for quantifying these epithelial-immune populations.

One of the limitations of this study was the selection of the control group. While this group was considered pathologically normal after careful review by physicians, the indication for endoscopy implies potential gastrointestinal disturbances. This is an inherent limitation as performing endoscopies on completely healthy children would not be ethically acceptable. Despite this, there is high confidence based on both diagnosis of the physician and the pathology evaluation that, at the minimum, we are comparing pediatric patients with and without Crohn’s disease in this analysis. Further chart review two years post sample collection also confirmed that none of the children in the control group have since been diagnosed with Crohn’s disease or any other inflammatory gastrointestinal diseases.

Another limitation is the small sample size. Pediatric Crohn’s disease is rare, with an annual incidence of 3–20 per 100,000^58^. In addition to this disease being rare, acquiring biopsies from this vulnerable population during remission presents additional challenges. In order to overcome these challenges, our study leveraged a subset of collected samples from the BEED study^26^. Due to the rarity of biopsy samples for children we were only able to obtain samples from 6 children with treated Crohn’s disease and 16 control children. All patients were undergoing treatment at University of Virginia Health System, which further limits the generalizability of these findings. This underlies the need for more open-source microbiome data. There are currently established research networks of patient-reported data from the Crohn’s and Colitis Foundation of America^59^. However, there is a lack of well-established national registries and biobanks for microbiome data related to Crohn’s disease.

Our data indicate that subtle dysbiosis of the small intestinal microbiota persists in pediatric Crohn’s patients despite resolved inflammation and repaired epithelial integrity. This implies that existing therapies are not sufficient to resolve Crohn’s disease and suggests dysbiosis as a therapeutic target. The efficacy of fecal microbiota transplantation (FMT) for Crohn’s disease further supports the importance of microbiota restoration as a part of treatment^60^. Recently, mycobiota have also been correlated with increased disease activity^61^. Thus, more extensive metagenomic studies to determine if recalcitrant dysbiosis in pediatric Crohn’s disease are warranted. Identifying specific species linked to pathogenesis, or conversely to sustained remission, could lead to novel microbiota-directed therapies. In addition, the identification of *Oribacterium* in patients with controlled Crohn’s disease suggests modification of the oral microbiome may be a unique therapeutic target. A greater understanding of the microbial drivers of relapse and remission could inform personalized treatment regimens based on patient-specific microbiota gaps.

### Supplementary Information

Supplementary Information.

## Data Availability

The datasets generated and/or analyzed during the current study are available in the dbGAP repository, PRJNA562573. Additional de-identified data supporting the findings of this study are available from the corresponding author upon request.
