# Supplementary material for: Persistent dysbiosis of duodenal microbiota in patients with controlled pediatric Crohn’s disease after resolution of inflammation
Source: Sci Rep. 2024 Jun 3;14:12668. doi: 10.1038/s41598-024-63299-y (PMC11148174; doi:10.1038/s41598-024-63299-y)
Supplement: Supplementary file 1 — Supplementary Information. [file 41598_2024_63299_MOESM1_ESM.pdf]

Persistent dysbiosis of duodenal microbiota in patients with controlled pediatric Crohn's disease after resolution of inflammation

Rebecca Pierce**[1], Ning-Jiun Jan**[1], Pankaj Kumar[2], Jeremy Middleton[3], William A. Petri[1], Chelsea Marie[1]*

1. Division of Infectious Diseases and International Health, Department of Medicine, University of Virginia School of Medicine, Charlottesville, VA, USA
2. Department of Biochemistry and Molecular Genetics, University of Virginia School of Medicine, Charlottesville, Virginia, USA
3. Division of Gastroenterology and Hepatology, Department of Medicine, University of Virginia School of Medicine, Charlottesville, Virginia, USA

*Corresponding author can be reached at csm8r@virginia.edu

** These two authors contributed equally to this work

SUPPLEMENTAL MATERIALS

Contents

Supplementary Methods

Cohort selection

Duodenal biopsy sample collection, RNA extraction, and RNA-sequencing

Supplementary Results

Table S1: Immunohistochemistry protein marker panel

Table S2: Flow cytometry panel

Table S3: Pediatric Crohn's disease showed deficiency and overabundance of specific bacterial taxa

Table S4: Additional descriptive statistics

Table S5: Differential gene expression in patients with controlled pediatric Crohn's disease compared to controls

Figure S1. Representative gating strategy for flow cytometry PBMC (A) and LPMC (B).

Figure S2. Gene expression profiles do not distinguish pediatric Crohn's disease patients from controls.

Supplementary Methods

Cohort Selection

An overview of the number of samples is shown in the flow chart. This trial was conducted as an ancillary analysis in a cohort of 90 patients enrolled under the control cohort arm of the BEED protocol[23]. This study has been7 patients had a confirmed diagnosis of CD and were undergoing treatment. For these patients, the study endoscopy visit was performed as part of routine clinical care to monitor treatment and disease. Samples from one CD patient were excluded because endoscopy revealed ongoing active disease. The final analysis included 6 patients with CD in remission (rmCD) and 16 patients with no pathogenic abnormality (NPA) upon endoscopy as a comparison group.

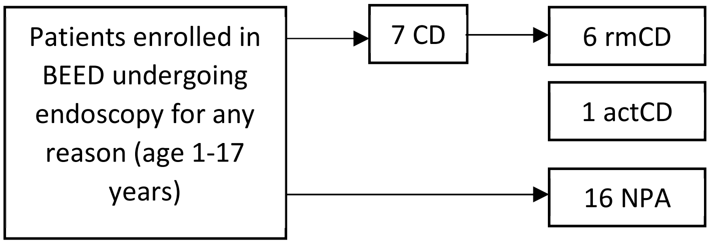

Duodenal biopsy sample collection, RNA extraction, and RNA-sequencing

Pinch duodenal biopsies obtained during endoscopy were immediately placed in allprotect (Qiagen) and stored at -80 C until extraction. RNA was prepared using the Qiagen AllPrep RNA/ DNA Kit. Ribosomal RNA was depleted using the rRNA depletion kit (NEB E6310). Libraries were prepared using the NEB ultradirectional library preparation kit 2.0 (NEB E7765) and indexed NEBNext Multiplex Oligos for Illumina(Dual Index Primers Set 1) (NEB E7600). Library size and purity were verified using an Agilent HS DNA bioanlyzer. Library concentration was measured by qPCR and high sensitivity Qubit. Libraries concentrations were normalized and 6 libraries were multiplexed per run. Diluted Libraries and PhiX controls were run on the Illumina Nextseq 500 in high output mode (400 Million reads, 2x75bp paired end, 150 cycles, 50 million reads per sample).

Raw reads were assessed by FASTQC (2) and mapped to the hg19 reference genome using HISAT2(3). RNAs were assembled, mapped to transcripts and quantified using STAR. Raw counts were processed using the bioconductor package DESeq2 v1.28.1 in R v 4.0.2 and

normalized using the DESeq algorithm. Genes with fewer than 10 counts across samples were prefiltered prior to differential expression analysis. Variance stabilizing transformation was applied to obtain normalized log2 gene expression values. Further quality control was performed using principal component analysis, boxplots, histograms and density plots. Differentially expressed genes were calculated using the Wald test in DESeq2 (4).

Supplementary Results

Table S1. Immunohistochemistry protein marker panel.

Biopsies were stained with various immunohistochemical protein markers below. Using these markers, the surface area staining for intraepithelial lymphocytes (IEL), lipocalin2 (LCN2), dual oxidase2 (DUOX2), tight junction protein 1 (ZO-1), and granzyme B (GZMB) was normalized by the epithelial area (positive for KRT18 staining).

Protein Marker	Biological Target
CD19	B lymphocytes
CD3	T lymphocytes
CD45	All leukocytes
CXCL10	Subsets of leukocytes
DEFA5	Paneth cells
DUOX2	Subsets of epithelial cells
GZMB	Subsets of leukocytes
KRT18	Epithelial cells
LCN2	Mucus epithelial cells and granulocytes
MKI67	Nuclei of cells in S phase
MUC2	Goblet cells
REG1B	Subsets of epithelial cells
SI	Brush border of epithelial cells
SLC15A1	Membranes of epithelial cells
SLC6A19	Membranes of epithelial cells
TJP1	Apical surface of epithelial cells

Table S2. Flow cytometry panel.

For analyzing the local tissue-specific immune cell populations, Cells were thawed and stained with fluorochrome-conjugated antibodies listed below. Samples were analyzed with the five-laser Cytek Aurora Borealis flow cytometer.

Immune Cell Marker	Fluorophore
Live/Dead	Zombie NIR
CD45	Qdot 800
CD3	Spark550
CD8	BV750
CD127	eFluor506
FoxP3	BV421
CD4	BV650
CD16	PE-Cy7
CD64	APC-fire 750
HLA-DR	BV785
CD11b	BV510
CD117	PerCP-Cy5.5
CD294	PE-Cy5
CD19/20	APC-Cy5.5
CD25	PE-Dazzle 594
TBET	BV711
CD45RA	BV570
CD56	AF700
CD14	Pacific Blue
GATA3	PE

Ki67	AF532
CD163	BV605
RORgT	AF647
gdTCR PerCP	EFlour 710

Table S3: Pediatric Crohn's disease showed deficiency and overabundance of specific bacterial taxa.

Differential community composition in the epithelial microbiota in Crohn's disease is characterized by increased *Prevotella* and *Oribacterium* and decreased *Actinobacillus* and *Streptococcus*. Table shows taxa level differences between pediatric Crohn's disease and control in epithelial-associated microbiota abundance. Differences with a $|\log_2\text{fold change}| > 6$ and $p < 0.05$ by the Wald test are displayed below.

Kingdom	Family	Genus	Species	log2 fold change	*p* value
Bacteria	*Streptococcaceae*	*Streptococcus*	NA	-6.0	0.02
Bacteria	*Prevotellaceae*	*Prevotella*	NA	7.5	0.02
Bacteria	*Pasteurellaceae*	*Actinobacillus*	NA	-6.8	0.03
Bacteria	*Lachnospiraceae*	*Oribacterium*	*Asaccharolyticum*	6.4	0.05

Table S4. Additional descriptive statistics.

Below is a descriptive statistical summary of all boxplots in the main text including alpha diversity, epithelial detachment score, immunohistochemistry, and flow cytometry. Values included are the median and interquartile ranges (IQRs) for both patients with Crohn's disease and controls.

			Controlled Crohn's disease		Control	
		Measure	Median	IQR	Median	IQR
Alpha diversity		Shannon (Biopsy)	3.56	0.64	3.79	0.77
		Simpson (Biopsy)	0.95	0.052	0.96	0.033
		Shannon (all samples)	3.33	0.70	3.70	0.68
		Simpson (all samples)	0.94	0.047	0.95	0.038
Epithelial detachment score			0.5	0	1	0.5
Immunohistochemistry		duox2	0.00076	0.0014	0.00045	0.0015
		LCN	0.00089	0.0031	0.00026	0.00049
		IEL	0.02	0.0075	0.025	0.023
		ZO-1	0.375	0.12	0.41	0.10
		GZMB	0.051	0.015	0.048	0.044
Flow Cytometry (Percentage of $CD45^+$)	PBMCs	PBMC T cell	80.68	5.43	78.13	8.14
		PBMC Th	48.82	1.98	40.59	10.97
		PBMC Th1	0.010	0.019	0.011	0.041
		PBMC Th2	0.079	0.018	0.088	0.095
		PBMC Th17	0.017	0.029	0.026	0.036
		PBMC Treg	0.0011	0.0029	0.0016	0.015
		PBMC $CD8^+$	23.04	9.41	22.62	6.31

Flow Cytometry (Percentage of $CD45^+$)		PBMC ILC1	0	0	0	5.43e-05
		PBMC ILC2	0.00030	0.00056	0.00043	0.0014
		PBMC ILC3	0.00025	0.0015	0.00064	0.0019
	LPMCs	LPMC T cell	9.81	5.78	11.33	6.11
		LPMC Th	0.93	1.07	0.78	0.77
		LPMC Th1	0.024	0.015	0.010	0.017
		LPMC Th2	0.0054	0.011	0.0025	0.0019
		LPMC Th17	0.015	0.013	0.012	0.015
		LPMC Treg	0.022	0.015	0.018	0.018
		LPMC $CD8^+$	5.57	4.45	8.84	6.18
		LPMC ILC1	0.077	0.065	0.042	0.029
		LPMC ILC2	0.037	0.047	0.027	0.076
		LPMC ILC3	0.11	0.14	0.11	0.13

Table S5: Differential gene expression in patients with controlled pediatric Crohn's disease compared to controls.

RNA-seq analysis did not identify any differentially expressed genes between the patients with controlled Crohn's disease and controls that met the criteria for significant differential expression (padj <0.05).

ENSEMBL ID	gene name	base mean	log2fold change	*p* value	padj	Gene Biotype
ENSG00000125845	BMP2	397.57	-0.78	3.13e-05	0.47	protein coding
ENSG00000270058	*NA*	7.40	-1.98	4.86e-05	0.47	*NA*
ENSG00000228232	GAPDHP1	4.12	-5.08	5.38e-05	0.47	processed pseudogene
ENSG00000137959	IFI44L	113.41	2.72	3.81e-05	0.47	protein coding
ENSG00000259479	SORD2P	20.64	-1.34	1.97e-04	0.999	transcribed unprocessed pseudogene
ENSG00000174353	STAG3L3	44.37	-0.98	2.76e-04	0.999	transcribed unprocessed pseudogene
ENSG00000226278	PSPHP1	28.61	-7.85	3.56e-04	0.999	unprocessed pseudogene
ENSG00000169245	CXCL10	25.14	-1.88	3.70e-04	0.999	protein coding
ENSG00000124249	KCNK15	53.51	-1.70	4.51e-04	0.999	protein coding
ENSG00000128040	SPINK2	14.60	-1.88	4.68e-04	0.999	protein coding

A

PBMCs

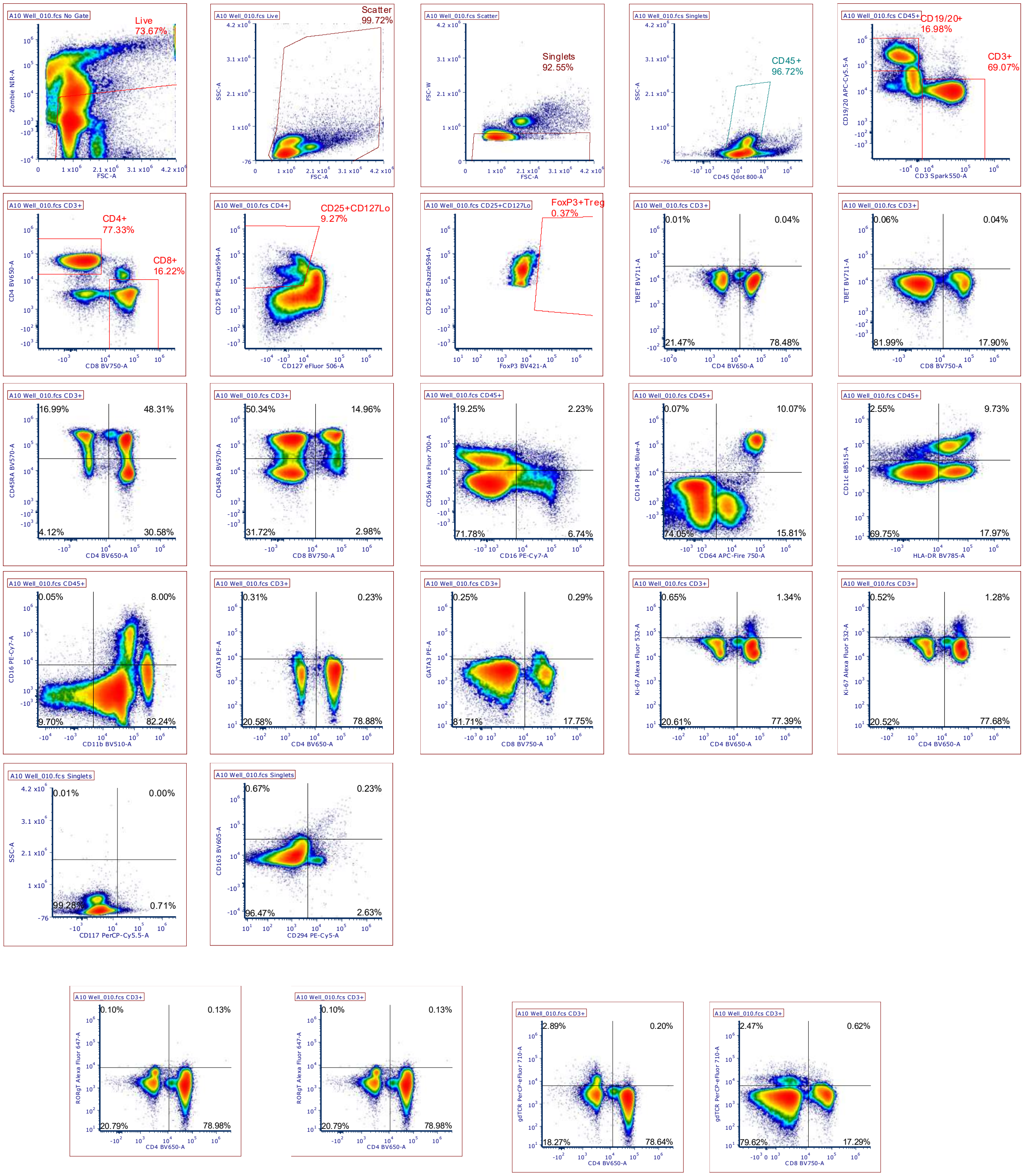

A10 Well_010.fcs No Gate
Live 73.67%
Zombie NIR-A
FSC-A
A10 Well_010.fcs Live
Scatter 99.72%
SSC-A
FSC-A
A10 Well_010.fcs Scatter
Singlets 92.55%
FSC-W
FSC-A
A10 Well_010.fcs Singlets
CD45+ 96.72%
SSC-A
CD45 Qdot 800-A
A10 Well_010.fcs CD45+
CD19/20+ 16.98%
CD3+ 69.07%
CD19/20 APC-Cy5.5-A
CD3 Spark550-A
A10 Well_010.fcs CD3+
CD4+ 77.33%
CD8+ 16.22%
CD4 BV650-A
CD8 BV750-A
A10 Well_010.fcs CD4+
CD25+CD127Lo 9.27%
CD25 PE-Dazzle594-A
CD127 eFluor 506-A
A10 Well_010.fcs CD25+CD127Lo
FoxP3+Treg 0.37%
CD25 PE-Dazzle594-A
FoxP3 BV421-A
A10 Well_010.fcs CD3+
0.01% 0.04% 21.47% 78.48%
TBET BV711-A
CD4 BV650-A
A10 Well_010.fcs CD3+
0.06% 0.04% 81.99% 17.90%
TBET BV711-A
CD8 BV750-A
A10 Well_010.fcs CD3+
16.99% 48.31% 4.12% 30.58%
CD45RA BV570-A
CD4 BV650-A
A10 Well_010.fcs CD3+
50.34% 14.96% 31.72% 2.98%
CD45RA BV570-A
CD8 BV750-A
A10 Well_010.fcs CD45+
19.25% 2.23% 71.78% 6.74%
CD56 Alexa Fluor 700-A
CD16 PE-Cy7-A
A10 Well_010.fcs CD45+
0.07% 10.07% 74.05% 15.81%
CD14 Pacific Blue-A
CD64 APC-Fire 750-A
A10 Well_010.fcs CD45+
2.55% 9.73% 69.75% 17.97%
CD11c BB515-A
HLA-DR BV785-A
A10 Well_010.fcs CD45+
0.05% 8.00% 9.70% 82.24%
CD16 PE-Cy7-A
CD11b BV510-A
A10 Well_010.fcs CD3+
0.31% 0.23% 20.58% 78.88%
GATA3 PE-A
CD4 BV650-A
A10 Well_010.fcs CD3+
0.25% 0.29% 81.71% 17.75%
GATA3 PE-A
CD8 BV750-A
A10 Well_010.fcs CD3+
0.65% 1.34% 20.61% 77.39%
Ki-67 Alexa Fluor 532-A
CD4 BV650-A
A10 Well_010.fcs CD3+
0.52% 1.28% 20.52% 77.68%
Ki-67 Alexa Fluor 532-A
CD4 BV650-A
A10 Well_010.fcs Singlets
0.01% 0.00% 99.28% 0.71%
SSC-A
CD117 PerCP-Cy5.5-A
A10 Well_010.fcs Singlets
0.67% 0.23% 96.47% 2.63%
CD163 BV605-A
CD294 PE-Cy5-A
A10 Well_010.fcs CD3+
0.10% 0.13% 20.79% 78.98%
RORgT Alexa Fluor 647-A
CD4 BV650-A
A10 Well_010.fcs CD3+
0.10% 0.13% 20.79% 78.98%
RORgT Alexa Fluor 647-A
CD4 BV650-A
A10 Well_010.fcs CD3+
2.89% 0.20% 18.27% 78.64%
gdTCR PerCP-eFluor 710-A
CD4 BV650-A
A10 Well_010.fcs CD3+
2.47% 0.62% 79.62% 17.29%
gdTCR PerCP-eFluor 710-A
CD8 BV750-A

B

LPMCs

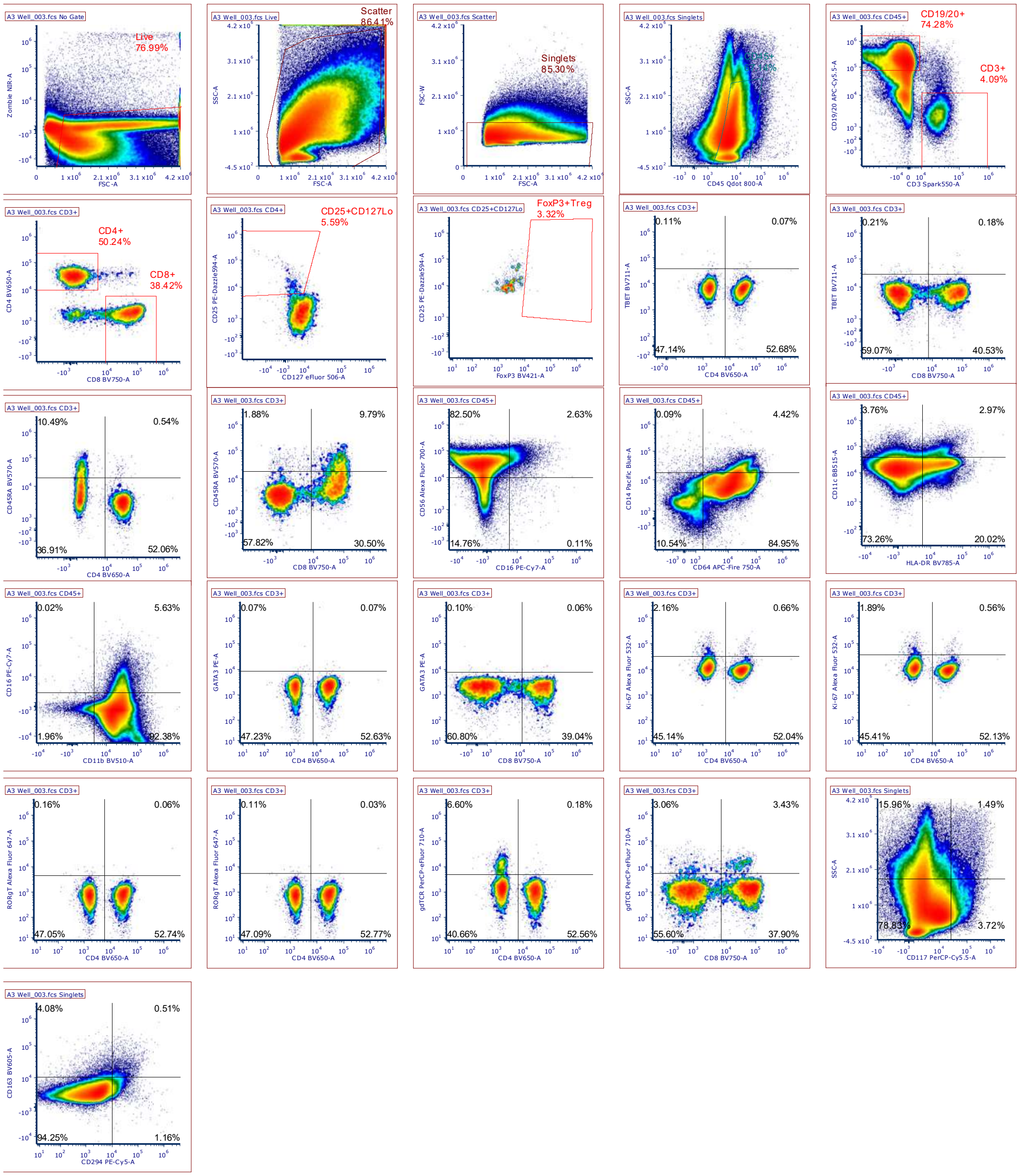

Figure S1. Representative gating strategy for flow cytometry PBMCs (A) and LPMCs (B).

Live dead gating was followed by a scatter gate and a single cell gate before determining specific cell populations.

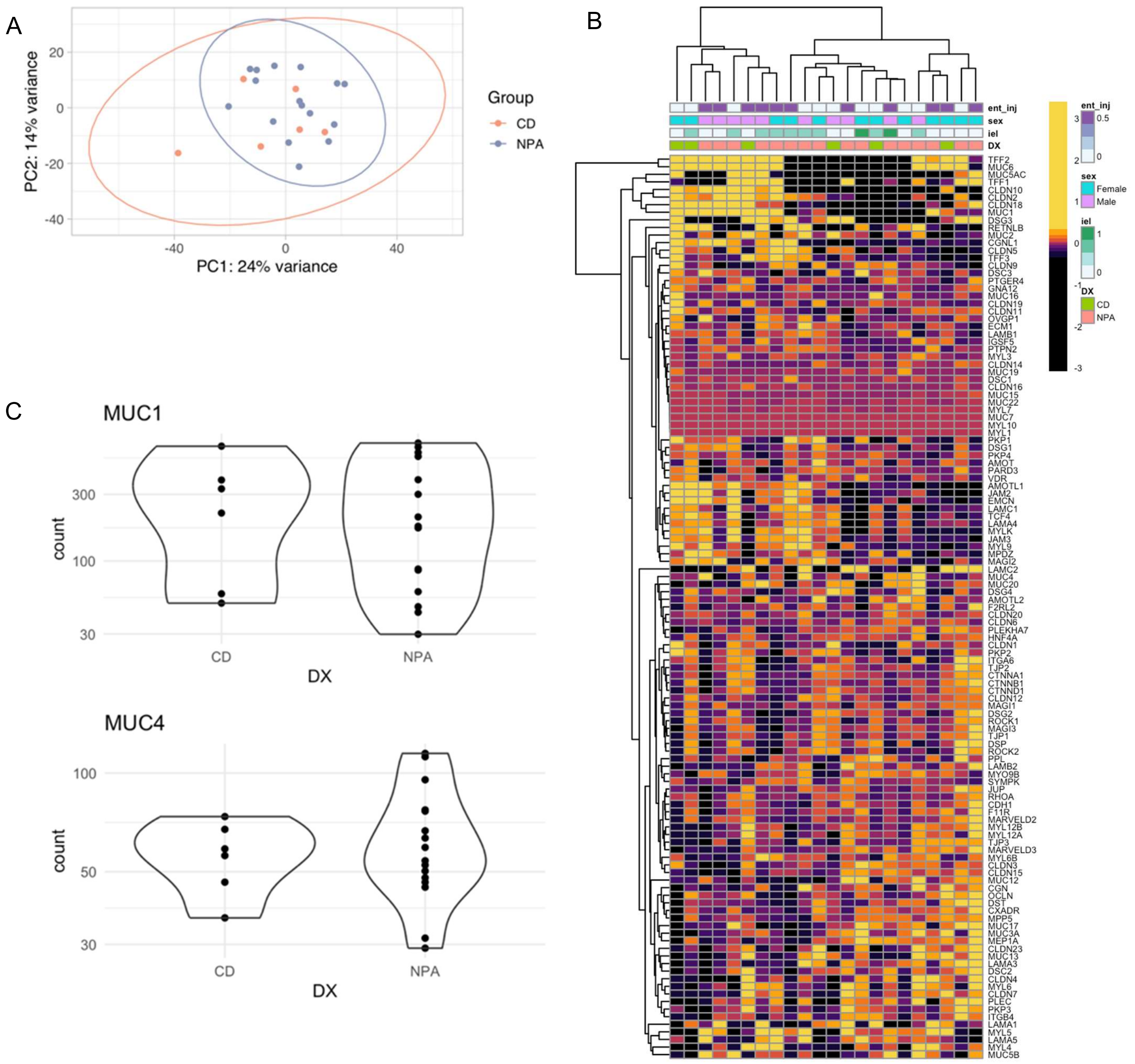

Figure S2. Gene expression profiles do not distinguish pediatric Crohn's disease patients from controls.

A. Principal component analysis based on RNA-seq gene expression data of patients with controlled Crohn's disease (CD) and control patients (NPA). No clear distinction in grouping between Crohn's disease patients and controls was observed. B. No apparent trends toward altered expression of epithelial integrity genes between Crohn's diesease and controls. C. *MUC1* and *MUC4* levels were similar in both cohorts.
